# Microbial Functional Responses in Marine Biofilms Exposed to *Deepwater Horizon* Spill Contaminants

**DOI:** 10.3389/fmicb.2021.636054

**Published:** 2021-02-25

**Authors:** Rachel L. Mugge, Jennifer L. Salerno, Leila J. Hamdan

**Affiliations:** ^1^Division of Coastal Sciences, School of Ocean Science and Engineering, University of Southern Mississippi, Ocean Springs, MS, United States; ^2^Department of Environmental Science and Policy, George Mason University, Fairfax, VA, United States

**Keywords:** biofilm, metagenome, microcosm, *Deepwater Horizon* oil spill, functional redundancy, microbiome

## Abstract

Marine biofilms are essential biological components that transform built structures into artificial reefs. Anthropogenic contaminants released into the marine environment, such as crude oil and chemical dispersant from an oil spill, may disrupt the diversity and function of these foundational biofilms. To investigate the response of marine biofilm microbiomes from distinct environments to contaminants and to address microbial functional response, biofilm metagenomes were analyzed from two short-term microcosms, one using surface seawater (SSW) and the other using deep seawater (DSW). Following exposure to crude oil, chemical dispersant, and dispersed oil, taxonomically distinct communities were observed between microcosms from different source water challenged with the same contaminants and higher Shannon diversity was observed in SSW metagenomes. *Marinobacter*, *Colwellia*, *Marinomonas*, and *Pseudoalteromonas* phylotypes contributed to driving community differences between SSW and DSW. SSW metagenomes were dominated by Rhodobacteraceae, known biofilm-formers, and DSW metagenomes had the highest abundance of *Marinobacter*, associated with hydrocarbon degradation and biofilm formation. Association of source water metadata with treatment groups revealed that control biofilms (no contaminant) harbor the highest percentage of significant KEGG orthologs (KOs). While 70% functional similarity was observed among all metagenomes from both experiments, functional differences between SSW and DSW metagenomes were driven primarily by membrane transport KOs, while functional similarities were attributed to translation and signaling and cellular process KOs. Oil and dispersant metagenomes were 90% similar to each other in their respective experiments, which provides evidence of functional redundancy in these microbiomes. When interrogating microbial functional redundancy, it is crucial to consider how composition and function evolve in tandem when assessing functional responses to changing environmental conditions within marine biofilms. This study may have implications for future oil spill mitigation strategies at the surface and at depth and also provides information about the microbiome functional responses of biofilms on steel structures in the marine built environment.

## Introduction

The marine built environment sustains the vast biodiversity of life present in the soft bottom habitat of the seafloor (Gage, 2004). Historic shipwrecks and other submerged structures, such as oil pipelines and drilling rigs, have the potential to transform into artificial reefs on the seabed, but marine biofilms are the biological foundation of these ecosystems. Pioneer microorganisms immediately initiate biofilm formation on submerged structures through surface adhesion, release of extracellular polymers, and recruitment of diverse microorganisms to the substrate. Once established, mature biofilms release chemical signals that serve as settlement cues for other micro- and macrofauna (Hadfield, 2011; Dobretsov and Rittschof, 2020). Exposure to aquatic pollutants, such as crude oil and dispersant, impact biofilm composition and function (Salerno et al., 2018; Mugge et al., 2019a, b), with potential downstream effects on the diversity of higher trophic level organisms recruiting to artificial reefs.

Following the 2010 *Deepwater Horizon* spill, oil plumes were detected at 1,000–1,300 m water depth at distances up to 16 km surrounding the 1,500-m wellhead until it was capped after 85 days (Valentine et al., 2014; Gao et al., 2015). An estimated 4–31% of total oil released from the wellhead was sequestered to the seafloor at estimated depths of 1,300–1,700 m on the continental slope, based on the detection of oil biomarkers in seafloor sediment (Valentine et al., 2014). To expedite bioremediation of the oil, an estimated total of 7 million liters of the chemical dispersant COREXIT EC9500A was applied at the surface and at the wellhead (Kleindienst et al., 2016). Within the acute spill footprint (∼16 km), where spill contaminants were deposited on the seafloor, a 5-cm-thick reddish-brown surface layer, high porosity (∼90%), and a microbiome dominated by bacteria with the functional potential for hydrocarbon degradation were observed in collected sediment cores (Hamdan et al., 2018). Sediment, biofilm, and water microbiomes from samples collected around historic shipwrecks within the acute spill footprint indicate that residual spill contaminants may induce changes to foundational biofilm microbiomes on artificial reefs, which may negatively impact preservation of these structures (Mugge et al., 2019a).

Access to the deep sea is limited, and therefore, biofilm formation on submerged structures and the impacts of environmental contaminants on biofilm composition and function are not well understood. Carbon steel disks (CSDs) are commonly used as biofilm recruitment surfaces in simulated experiments since the majority of marine infrastructure is constructed from carbon steel (McBeth et al., 2011). Salerno et al. (2018) examined biofilm response to spill contaminants by placing CSDs in microcosms filled with surface seawater (SSW) and amended with environmentally relevant concentrations of crude oil, dispersant, and dispersed oil to discover how submerged metal infrastructure may be compromised by oil spills. Mugge et al. (2019b) replicated this study using deep seawater (DSW), and the results from both studies reveal that exposure to spill contaminants immediately induces changes to the biodiversity and function of bacteria inhabiting marine biofilms. Investigating the metagenomes sequenced from the same biofilm samples may give insight into the functional redundancy of marine biofilms exposed to spill contaminants.

Functional redundancy is the ecological theory that taxonomically distinct species are capable of similar metabolic roles within communities and ecosystems (Rosenfeld, 2002); this theory has been extended to microbial assemblages (Allison and Martiny, 2008; Burke et al., 2011). From an evolutionary perspective, functional redundancy benefits ecosystems by allowing the diversity and abundance of species to be interchanged with little or no impact to the function of the ecosystem. However, the generality of functional redundancy may not hold true for microbial assemblages under changing environmental conditions and altering the composition of a microbiome may also alter its functions (Fetzer et al., 2015; Galand et al., 2018).

Marine microorganisms play vital roles in global carbon and nutrient cycling, feedback mechanisms, and organic matter fluxes to the seafloor (Bienhold et al., 2016). Sequencing technology has provided insight into the diversity, abundance, and functional capabilities of deep-sea bacterial communities, which are largely unclassified. In comparison to seawater and sediment habitats, marine biofilm microbiomes are distinct due to surface associations that increase the relative abundance of novel taxa and functional genes (Zhang et al., 2019). Additionally, environmental parameters such as pH, dissolved oxygen, light, and nutrient availability play major roles in determining the structure of marine biofilm microbiomes (Orland et al., 2019). While microbial communities are sensitive to disturbances, they can also be resistant and resilient to environmental changes (Allison and Martiny, 2008). The impacts of anthropogenic environmental disturbances on the diversity and functional redundancy of marine biofilms, however, is understudied.

Studying marine biofilm recruitment, and response to contaminants, over time may provide insight into how microbiomes on built structures respond to environmental disturbance. Accordingly, our study quantified changes in developing marine biofilm microbiomes following exposure to *Deepwater Horizon* oil spill contaminants, using metagenomics to determine if functional similarity occurs between developing biofilms sourced from SSW and DSW. We hypothesized that biofilms recruited to carbon steel will harbor distinct microbial taxa due to different seawater source populations but will exhibit similar functional responses to contaminant exposure.

## Materials and Methods

### Laboratory Microcosms

Two separate laboratory experiments were carried out using different source water to recruit microbiomes to C1020 mild CSDs to simulate biofilm growth on built marine infrastructure. CSDs (Metal Samples Company, Munford, AL, United States) measured 1.59 cm in diameter and 0.016 cm thick with a mill finish and were mounted using EpoThin2 epoxy (Buehler, Lake Bluff, IL, United States) with one face exposed, following the protocol of Lee et al. (2004, 2005). Inside microcosm tanks, CSDs were housed in short towers (16.51 cm outer diameter by 27.94 cm high) constructed from polyvinyl chloride (PVC) pipe (Commercial Industrial Supply, Rockhill, SC, United States) and secured using 100% waterproof silicon sealant (Silicone Max, all purpose). Mugge et al. (2019b) provides further description of the experimental design. To collect biofilm samples, CSDs were sacrificed biweekly (*n* = 2) and stored upright in sterile plastic collection cups at −80°C. Week 2 samples were collected before contaminant additions of crude oil, dispersant, and dispersed oil to microcosms, and all other samples were collected following exposure to contaminants.

For the SSW experiment, water was collected from the US Naval Research Laboratory Key West pier (Key West, FL, United States) and stored for 1 week in a cold room maintained at 4°C (±0.4°C). Four, 25-L experimental tanks were each filled with 16 L of seawater and kept covered to exclude ambient light, and on stir plates for continuous water circulation. Salinity (37–38 PSU), temperature (3.4–4.4°C), and dissolved oxygen (86.5–97.6%) were measured weekly for 16 weeks (Supplementary Table 1). After a 2-week acclimation period, each of the four tanks were amended with a single contaminant, as follows: 1 control tank (no contaminant), 1 oil tank (Louisiana sweet crude oil, 5 mg/L final concentration), 1 chemical dispersant tank (Corexit EC9500, 0.05 mg/L final concentration), and 1 dispersed oil tank (5 mg/L crude oil + 0.05 mg/L dispersant final concentration). Contaminant concentrations were calculated from a review of published data and from data obtained from the NOAA Office of Response and Restoration’s Environmental Response Management Application (ERMA) database.^1^

For the DSW experiment, water was collected from ∼10 m above the seafloor at the *Anona* shipwreck in the Viosca Knoll lease area in the Gulf of Mexico at an approximate depth of 1,200 m using twelve 12-L Niskin bottles attached to a conductivity, temperature, and depth (CTD) rosette on R/V *Point Sur* cruise PS17-26. Additional site information is provided in Hamdan et al. (2018). Seawater was collected into 13 autoclaved, 20-L polycarbonate carboys and stored at 4°C (±0.5°C) in a cold room. Each of the 12 experimental tanks (three replicate tanks for each of three contaminants, and three replicate control tanks) were filled with 16 L of seawater and maintained in the cold room, covered to exclude ambient light and on stir plates set to ∼620 rpm for continuous circulation. Salinity (35–39 PSU), temperature (4.8–5.6°C), and dissolved oxygen (77.3–94.8%) were measured weekly during the 14-week experiment (Supplementary Table 1). Water-accommodated fractions (WAFs), which have been implemented in aquatic toxicity studies, were used to simulate oil spill conditions in the tanks (Forth et al., 2017). Following 2 weeks of acclimation, replicate (*n* = 3) tanks were amended with crude oil using a high-energy water-accommodated fraction (HEWAF, 80 mL/tank; 5 mg/L final concentration of oil), diluted chemical dispersant (Corexit EC9500, 0.05 mg/L final concentration), dispersed oil using a chemically enhanced water-accommodated fraction (CEWAF, 80 mL/tank; 5 mg/L final concentration of oil and 0.05 mg/L final concentration of Corexit), or no treatment (control). HEWAF and CEWAF were made following the protocols as previously described (Mugge et al., 2019b).

### DNA Extraction and Quantitation

Biofilms, which include corrosion products, were removed from CSDs and collected into pre-weighed lysing matrix E tubes for DNA extraction (MP Biomedicals, LLC, Santa Ana, CA, United States) following a modified version of the manufacturer’s protocol of the BIO 101 FastDNA Spin Kit as previously described (Hamdan et al., 2013). Prior to DNA extraction from corrosion biofilms, bovine serum albumin (Promega, Madison, WI, United States) was added as a chelating agent to prevent inhibition of PCR amplification by iron ions (Salerno et al., 2018; Mugge et al., 2019b). Extracted DNA (150 μL) was stored frozen at −80°C. Total extracted genomic DNA (10 μL) was quantitated on a Qubit 2.0 Fluorometric Quantitation system (Invitrogen, Carlsbad, CA, United States) and a NanoDrop spectrophotometer (Thermo Fisher Scientific, Waltham, MA, United States) was used to assess purity. 16S rRNA amplicon sequence taxonomy data and metagenome data were obtained from the same samples from each respective microcosm. The metagenome data are presented in this study while the 16S rRNA data are published elsewhere (Salerno et al., 2018; Mugge et al., 2019b).

### Metagenome Analysis

Prior to metagenome sequencing at the Integrated Microbiome Resource (IMR) facility at Dalhousie University (Halifax, Nova Scotia, Canada), replicate samples from the DSW microcosm were pooled to minimize cost and for comparability to SSW metagenomes. DSW samples included one pooled sample from week 2 prior to contaminant addition, which served as the pretreatment sample, and pooled samples from replicate contaminant tanks for weeks 4, 8, 12, and 14 for a total of 17 DSW metagenomes. Sample pooling was based on statistical analysis of Shannon diversity and total assigned reads from replicate 16S rRNA gene amplicon sequences presented elsewhere (Mugge et al., 2019b; Supplementary Table 2). The SSW microcosm did not have replicate tanks and thus all samples were sequenced and only metagenomes from weeks 2, 4, 8, 12, and 14 were included for comparability with DSW metagenomes. Metagenome Libraries were prepared with the Nextera XT Library Preparation Kit (Illumina Inc.), a PCR-based library preparation approach, modified to use the Just-a-Plate^TM^ 96 PCR Purification and Normalization Kit (Charm Biotech) as described in Salerno et al. (2018). Sequencing was performed on an Illumina NextSeq 550, generating 150-bp, paired-end sequences. Metagenome sequences were submitted to the NCBI SRA under BioProject PRJNA623475.

### Metagenome Bioinformatics Analysis

Metagenomes were analyzed in accordance with workflows available in the Microbiome Helper repository (Comeau et al., 2017). Raw sequences were quality filtered (Andrews, 2010), assembled into paired-end reads (Zhang et al., 2013), trimmed (Bolger et al., 2014), and screened for contaminants (Langmead and Salzberg, 2012). MetaPHlAn2 was used to assign taxonomy based on unique clade-specific marker genes that unambiguously assign reads to microbial clades (Truong et al., 2015). OTU abundance was extracted from MetaPHlAn2 data, averaged by treatment within each microcosm, and relative abundance bar charts were created using Microsoft Excel, and excluded any taxonomic group that represented less than 2% of the total population per treatment. The relative abundance of all taxa at the OTU level for both experiments across all time points is included in Supplementary Table 3. HUMAnN2 (Franzosa et al., 2018) was used to profile gene families based on the UniRef90 database, and these features were renormalized to copies per million (CPM), and regrouped and renamed by using the Kyoto Encyclopedia of Genes and Genomes to assign KEGG orthologs (KOs; Kanehisa and Goto, 2000).

### Statistical Analysis

Taxa and gene abundance data from all SSW week 2 samples (*n* = 4) were each pooled and averaged as a SSW pretreatment sample to create an analogous comparison with the DSW pretreatment sample. Species-level annotations were extracted from the MetaPHlAn2 results of SSW and DSW microcosms, and an operational taxonomic unit (OTU) table was imported into PRIMER (v.6.13, PRIMER-E Ltd., Plymouth, United Kingdom). Functional profiles of metagenomes were analyzed using HUMAnN2, which resulted in abundance tables of gene families. The default output unit from HUMAnN2, reads per kilobase (RPK), accounts for gene length but was renormalized to CPM to account for sequencing depth using the humann2_renorm_table script. Following renormalization, a combined gene abundance table for SSW and DSW microcosms was imported to PRIMER. For taxonomy and functional profiles, Bray–Curtis dissimilarities were calculated, hierarchical clustering (CLUSTER) illustrated similarity based on the group average linkage, and non-metric multidimensional scaling (NMDS) plots were constructed to visualize community and functional differences in three-dimensional space. Following a permutational analysis of variance (PERMANOVA) main test on each profile, separate pairwise PERMANOVA tests were run on the interaction term “microcosm × treatment” to test for significant differences between the same treatments within each microcosm. Shannon diversity of both taxonomy and functional profiles was calculated using Quantitative Insights Into Microbial Ecology (QIIME). Assigned reads were correlated with observed OTUs from all metagenomes, and Excel was used to calculate the correlation coefficient, *t* score, and *p* value.

To observe functional responses to treatments, pretreatment samples were excluded in downstream functional comparative analyses. Mapping of the merged HUMAnN2 gene families file of all metagenomes to KOs resulted in 57% unmapped reads and 30% ungrouped reads, leaving 13% of reads mapped to KOs. These KOs were renormalized by CPM, averaged by treatment for each microcosm, and then ranked by overall abundance. Averaged values were input to PRIMER to visualize major differences in functional profiles by running a CLUSTER analysis. A similarity percentage (SIMPER) analysis was run to determine functional categories driving similarities and differences between metagenomes. The top 20% of assigned reads (top 149 abundant KOs) were assigned to a functional hierarchy using the hierarchical structure of the KEGG Brite database. Each hierarchy classification was summed and a bar chart was created using Microsoft Excel. To interrogate the dataset for functional similarity, MaAsLin2 (Mallick et al., 2017) was used to build a generalized linear model using a minimum abundance threshold of 0.001 and a significance of *p* < 0.05. This model was used to associate source water metadata with control and treatment groups and yielded an output of significantly different genes per treatment between SSW and DSW experiments based on relative abundance. Percentages of significant genes were calculated from filtered data and outputs of the model within MaAsLin2. The output of the analysis is the percentage of genes with a statistically significant differential abundance pattern, and thus, a higher percentage illustrates dissimilarity and a lower percentage corresponds to greater similarity.

## Results

**FIGURE 1:**
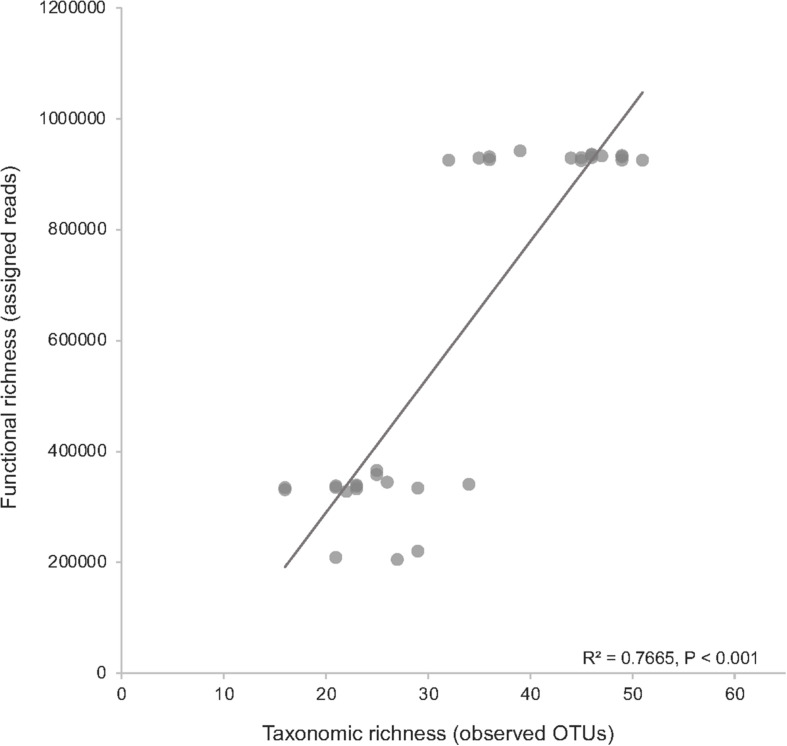
Comparison of community richness against the functional richness in metagenome samples. Taxonomic richness was measured as observed OTUs, and functional richness was measured as the number of assigned reads to KEGG orthologs (KOs) from all surface seawater (SSW) and deep seawater (DSW) metagenomes. The top cluster of data points are all SSW samples, while the bottom cluster are all DSW samples.

The SSW experiment had a larger number of observed OTUs (average 38) across all treatments compared to the DSW experiment (average 24) (Supplementary Table 2). Shannon diversity was greater, on average, in the SSW experiment (4.01), with the greatest average diversity in the oil treatment (4.17). Across all treatments, SSW metagenomes had a greater number of total assigned reads from gene families to KOs (900,000–1,000,000) as compared to DSW metagenomes (200,000–300,000) (Supplementary Table 2). Comparing taxonomic richness (observed OTUs) against functional richness (assigned reads to KOs) revealed a significant positive correlation (*R*^2^ = 0.77, *P* < 0.001) in both DSW and SSW metagenomes (Figure 1).

**FIGURE 2:**
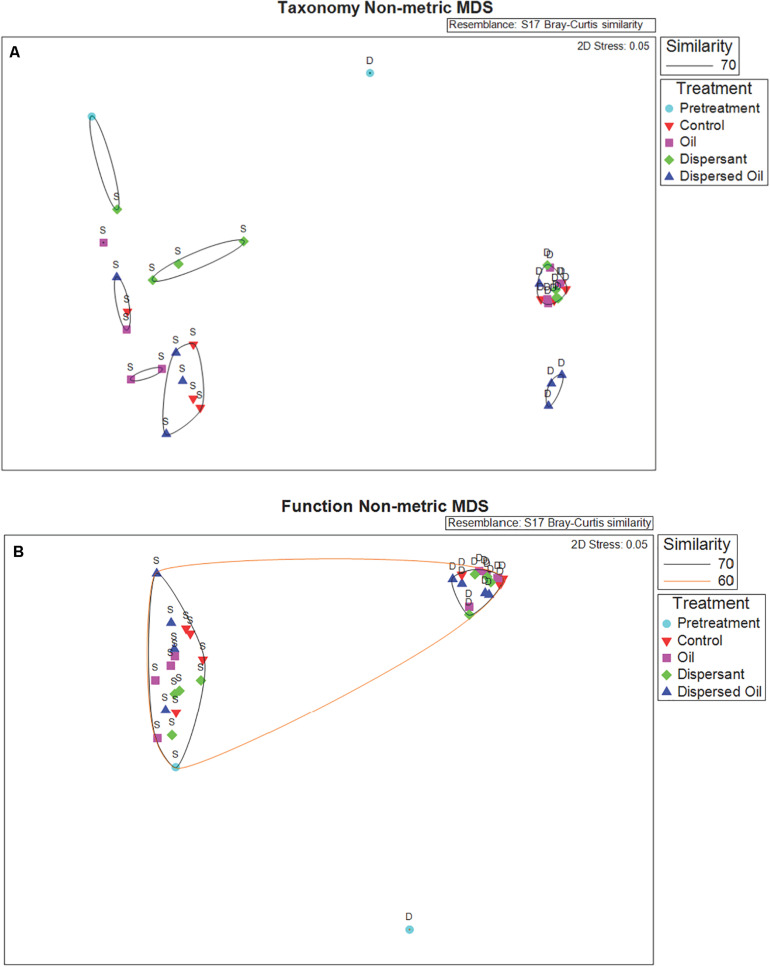
**(A)** Non-metric multidimensional scaling (NMDS) plot of Metaphlan2 species relative abundances of taxa and **(B)** Humann2 results of KEGG orthologs (KOs) in copies per million (CPM), which visualize differences in community structures (taxonomy NMDS) and functional profiles (function NMDS), respectively. Data obtained from surface seawater (SSW) and deep seawater (DSW) metagenomes based on Bray–Curtis dissimilarity matrices calculated within PRIMER. Icons represent metagenome samples color coded by treatment: pretreatment samples in light blue, control samples in red, oil samples in pink, dispersant samples in green, and dispersed oil samples in dark blue. Icons are labeled by source water used for the two separate experiments: SSW (S) and DSW (D). Samples are grouped at 60% (orange ellipse) and 70% (black ellipses) similarity based on respective cluster analyses.

An NMDS plot based on Bray–Curtis dissimilarities of taxa abundance data revealed separation between communities in the SSW and DSW experiments across all treatments, highlighting distinct microbial communities throughout the course of the experiments (Figure 2A). Although taxonomy in the DSW pretreatment sample displayed high separation from all other samples, the SSW pretreatment sample clustered at 70% similarity with one SSW dispersant sample. The cluster of DSW samples is visualized in an NMDS subset in Supplementary Figure 1A. A pairwise PERMANOVA comparing taxonomy data in the same treatments in SSW and DSW experiments returned statistically significant *p* values (*P* < 0.05), with control and dispersant communities exhibiting highest percent similarities (10.55%, 10.33%) (Supplementary Table 4).

An NMDS ordination revealed high separation between SSW and DSW metagenomic functional profiles based on Bray–Curtis dissimilarity of genes (Figure 2B). Similar to taxonomy, the DSW pretreatment sample separated from all other samples. An NMDS subset of all other DSW samples can be found in Supplementary Figure 1B. The SSW pretreatment sample clustered with all other SSW samples at 70% similarity. A pairwise PERMANOVA comparing genes from the same treatments across experiments returned statistically significant *p* values (*P* < 0.05) for all treatment comparisons (Supplementary Table 5). Average similarity ranged from 61.73% between control samples and 64.33% between dispersed oil samples.

**FIGURE 3:**
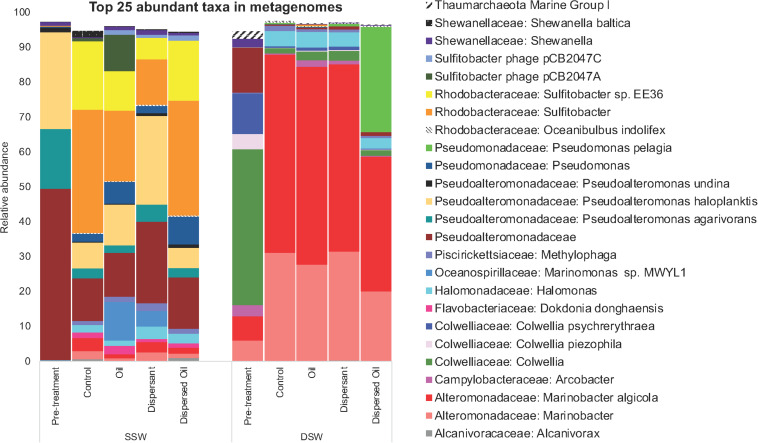
Relative abundance of OTUs at the species level extracted from Metaphlan2 analysis of biofilm metagenomes. Each bar reflects the average (*n* = 4) of all time points across each treatment for surface seawater (SSW) and deep seawater (DSW) microcosms. The top 25 abundant taxa are displayed in the figure, and taxa accounting for <2% of all sequences were excluded from the plot.

The SSW pretreatment community was dominated by an unclassified OTU in the Pseudoalteromonadaceae family while the DSW pretreatment community was dominated by *Colwellia* (Figure 3). *Sulfitobacter* were highly abundant (13–36%) in all SSW treatment communities in contrast to low abundance (0.3%) in the SSW pretreatment community. Two *Marinobacter* OTUs greatly increased in abundance in all DSW control and treatment communities as compared to pretreatment, while *Colwellia* decreased. Other OTUs that contributed to differences in pretreatment communities included *Pseudoalteromonas haloplanktis* and *Pseudoalteromonas agarivorans* in the SSW pretreatment and *Colwellia psychrerythraea* and *Marinobacter* in the DSW pretreatment. The DSW control and treatment communities were dominated by *Marinobacter algicola*, while *Sulfitobacter* was dominant in SSW control and treatment communities except for dispersant communities, which revealed *P. haloplanktis* to be most abundant. The DSW dispersed oil community revealed a high abundance of *Pseudomonas pelagia* compared to other DSW communities, and *Arcobacter* was more abundant in DSW oil and dispersant communities as compared to DSW control and dispersed oil communities. All SSW communities increased in diversity compared to pretreatment, with *Marinomonas* present only in SSW oil and dispersant communities, and a decreased abundance of Pseudomonadaceae and *Pseudomonas agarivorans*.

**FIGURE 4:**
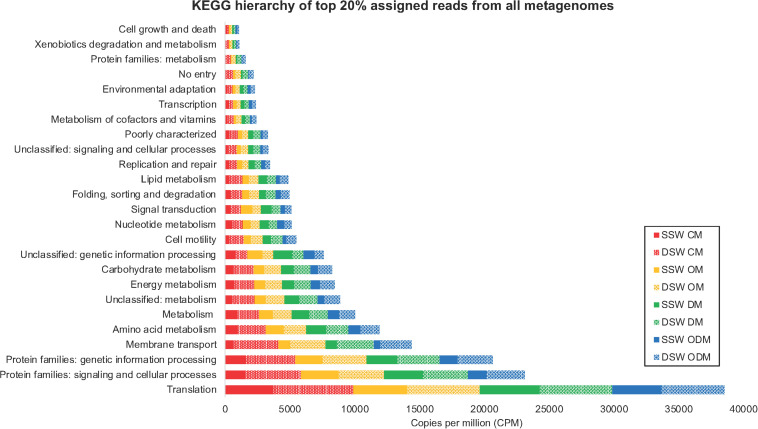
Stacked bar chart displaying KEGG hierarchy of top 149 abundant KEGG orthologs (KOs) accounting for 20% of assigned reads in averaged biofilm metagenomes. In sample names, SSW, surface seawater; DSW, deep seawater; CM, control microcosm; OM, oil microcosm; DM, dispersant microcosm; and ODM, dispersed oil microcosm.

**FIGURE 5:**
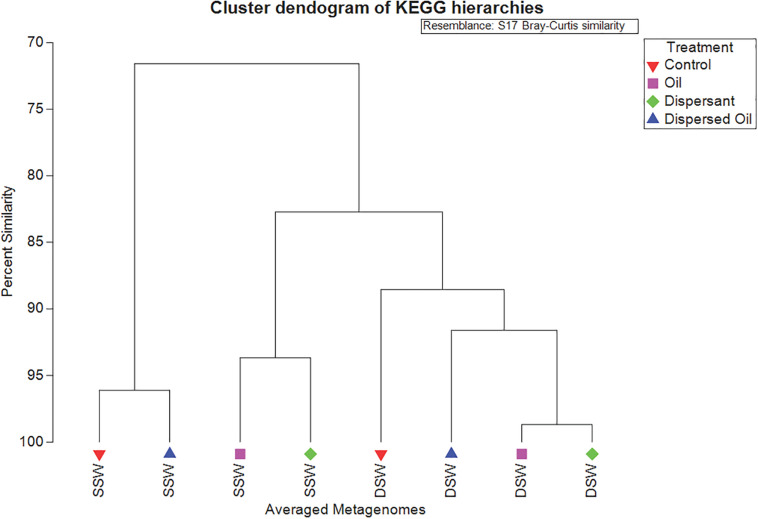
Similarity CLUSTER dendrogram analyzed in PRIMER based on Bray–Curtis dissimilarity of all averaged metagenomes showing percent similarities of the group averages calculated from KEGG hierarchies in copies per million (CPM). In sample names, SSW is surface seawater metagenomes and DSW is deep seawater metagenomes. Samples are color coded by treatment: control metagenomes are red triangles, oil metagenomes are pink squares, dispersant metagenomes are green diamonds, and dispersed oil metagenomes are dark blue triangles.

Using the KEGG Brite hierarchical structure to map individual KOs to functional categories (Supplementary Table 6) yielded 25 functional hierarchies consisting of the top 149 abundant KOs (Supplementary Table 7) in all metagenomes, which account for 20% of assigned reads (Figure 4). A CLUSTER analysis (Figure 5) was used to illustrate KEGG hierarchy similarity of the most abundant KOs from the different treatments and experiments. This shows that all treatments were 70% similar to each other. At the 80% similarity threshold, all metagenomes from the DSW experiment and two from the SSW experiment (oil and dispersant) clustered together, while the control and dispersed oil treatments from SSW grouped apart. In both experiments, oil and dispersant treatment metagenomes were greater than 90% similar to each other.

A SIMPER analysis revealed that membrane transport was the highest contributing category to overall differences between SSW and DSW, while translation drove similarities within each experiment. Translation was also the most abundant category overall and consisted of 30 KOs corresponding to large and small subunit ribosomal proteins, with higher reads in the DSW microcosm. Within this subset, nine categories of metabolism were annotated and the DSW microcosm had higher reads overall, with the DSW control being the highest. Membrane transport and cell motility KOs, which included chemotaxis and flagellar proteins, also had higher reads in the DSW microcosm compared to the SSW microcosm, with the highest abundance in the DSW control and the lowest abundance in the SSW dispersed oil. Lipid metabolism KOs had higher abundance in both SSW and DSW oil and dispersant metagenomes, which may have contributed to differences in SSW control and dispersed oil metagenomes compared to SSW oil and dispersant metagenomes. Signal transduction KOs had the highest abundance in SSW oil and dispersant metagenomes. Replication and repair KOs had similar abundances across both SSW and DSW oil and dispersant treatments, but had greater abundance in the DSW control.

Using MaAsLin2 to create a model to associate metadata with relative abundances revealed genes with a statistically significant (*P* < 0.05) differential occurrence pattern within the control group and each treatment group (Table 1). Output from analysis of filtered data revealed control metagenomes had the highest percentage (91.45%) of significant genes, indicating that this group had the most dissimilar response between SSW and DSW experiments. Oil metagenomes revealed that 49.03% significant genes and dispersant metagenomes had 49.44% significant genes. The most similar functional response was in dispersed oil metagenomes, with 45.99% significant genes. Lower percentages of significant genes observed in all treatment comparisons as compared to the control comparison indicates a more dissimilar functional response in the control groups from both experiments, and a more similar functional response in treatment groups from both experiments, specifically the dispersed oil metagenomes.

**TABLE 1:** Statistically significant (*P-adj.* < 0.05) gene associations between surface seawater (SSW) and deep seawater (DSW) experiments, grouped by treatment.

Treatment pairwise comparison	All gene associations (MaAsLin2)	Significant* gene associations	Percent significant* genes (%)	Taxa Shannon diversity (avg)	Function Shannon diversity (avg)
SSW CM (4), DSW CM (4)	1030	942	91.45	3.74	2.64
SSW OM (4), DSW OM (4)	1034	507	49.03	3.91	2.76
SSW DM (4), DSW DM (4)	1246	616	49.44	3.81	2.86
SSW ODM (4), DSW ODM (4)	711	327	45.99	3.90	2.35

*P-adj. < 0.05. Following filtering and normalization in MaAsLin2, significant gene associations were determined based on differences in relative abundances of gene (KEGG ortholog) copies between SSW and DSW experiments. Treatments abbreviated as follows: CM, control microcosm; OM, oil microcosm; DM, dispersant microcosm; ODM, dispersed oil microcosm.

## Discussion

This study investigated the compositional and functional responses of marine biofilms, formed from two different source populations, on carbon steel following exposure to crude oil, chemical dispersant, and dispersed oil. Previous studies indicate that marine biofilm formation and recruitment are linked to environmental parameters, including pH, temperature, radiation, and dissolved oxygen (Lee et al., 2014; Toyofuku et al., 2016; Orland et al., 2019). Changes in these parameters can potentially influence the community composition of mature marine biofilms in shallow water, although few studies have examined marine biofilms in the deep ocean. For this study, biofilm microbiomes were hypothesized to form taxonomically distinct communities when exposed to the same experimental treatments, because the source of the microbiomes originated from different environmental settings (surface water vs. deep-sea water). It was further hypothesized that the microbiomes would have similar functional responses, especially in the formative phases of the biofilms on the same surfaces under the same conditions. This was hypothesized based on evidence from previous studies of marine biofilms that detail the necessary conditions and phases of initial formation (Kaplan, 2010; Grzegorczyk et al., 2018). SSW microorganisms isolated from Key West, FL, initially had access to sunlight, warm temperatures, and more organic matter prior to experimentation, as opposed to DSW microbial communities sourced from the *Anona* shipwreck site (∼1,200 m), where pressure, absence of light, and cold (4°C) water temperatures were the prevailing *in situ* conditions. In support of testing these hypotheses, a side-by-side comparison of metagenome taxonomy profiles revealed SSW metagenomes harbored more observed OTUs and a greater average alpha diversity compared to DSW metagenomes. The microbial communities between SSW and DSW microcosms were distinct in pretreatment samples and remained distinct across time and different treatments.

Community analysis at a finer resolution revealed the OTUs contributing to community differences (Figure 3). Observations of different community structures between SSW and DSW experiments support the hypothesis that biofilms from two different source water populations will harbor distinct microbial taxa. The pretreatment biofilm communities for the SSW and DSW experiments contained some overlapping taxa, but as expected, given the different locations they were derived from, they contained distinct communities. A dramatic shift in community structure during the experiments (weeks 4–14) was evident in both SSW and DSW, and suggests that these early stage biofilms underwent a post-settlement maturation process (Salta et al., 2013). For the duration of the experiments, however, the DSW and SSW communities remained taxonomically distinct from each other, even though they were held under the same experimental conditions.

Rhodobacteraceae are known to be key members in initial marine biofilm formation (Elifantz et al., 2013; Kviatkovski and Minz, 2015; Dang and Lovell, 2016), and their prevalence in SSW control and treatment groups provides evidence of this process, even in the presence of contaminant addition. However, their lack of abundance in the DSW experiment indicates that other organisms must be involved in this process.

DSW metagenome communities also experienced a shift in community structure; while *Colwellia* dominated the pretreatment community, control and treatment groups during the experiment were dominated by *M. algicola* and another *Marinobacter* OTU, indicating a shift from free-floating to surface-attached bacterial communities (Grimaud et al., 2012; Mounier et al., 2014). While not as abundant, these same two OTUs appeared in the SSW experiment and combined were 2–6% of the total community. The genus *Marinobacter* is widespread in the deep ocean, and known for its capabilities of alkane degradation and EPS production (Handley and Lloyd, 2013; Gao et al., 2015; Arnosti et al., 2016; McKay et al., 2016; Hu et al., 2017). The latter capability (EPS production) potentially signals their involvement in initiating or maintaining marine biofilms. Others report differential abundances of *Marinobacter* and Rhodobacteraceae in comparative studies of biofilms formed offshore vs. inshore (Lema et al., 2019) and support the concept of taxonomically distinct communities with functionally redundant roles during this experiment.

Even though source populations were exposed to the same contaminants under the same environmental conditions, both experiments exhibited unique community responses during the experiments. Tremblay et al. (2017) showed that *Marinobacter* abundance increased with exposure to dispersed oil. In the current study, the abundance of *Marinobacter* declined in the DSW dispersed oil treatment, and both the SSW oil and dispersed oil treatment. Another prior study showed that some *Marinobacter* also declined in the presence of chemical dispersants under laboratory conditions (Kleindienst et al., 2015b), suggesting that both oil and chemical dispersants impact the abundance and function of natural oil-degrading and EPS-producing bacteria in the marine environment.

The DSW dispersed oil community was also enriched with by *P. pelagia*—a psychrotolerant bacterium (Hwang et al., 2009) that has recently been suggested to degrade hydrocarbons in subarctic sediments (Gontikaki et al., 2018). *P. haloplanktis*, another cold adapted bacterium (Qi et al., 2019) with reported hydrocarbon degradation capabilities under laboratory conditions (Chronopoulou et al., 2015), was observed in elevated abundance in the SSW oil and dispersant treatments, relative to the control group. *Colwellia* OTUs, also endemic to cold and deep marine environments and known to degrade hydrocarbons (Techtmann et al., 2016), were dominant in the oil plume during the *Deepwater Horizon* spill (Valentine et al., 2014). *Colwellia* metagenomes, from three *C. psychrerythraea* strains isolated from a deep-sea basin, have the ability to adapt to local conditions, degrade chemical dispersants, and produce EPS for biofilm formation (Techtmann et al., 2016). The abundance of *Colwellia* OTUs in all DSW biofilms, specifically OTUs affiliated with C. *psychrerythraea* in oil and dispersant treatments, suggests that they too may have roles in responding to contaminant exposure in biofilm communities (Kleindienst et al., 2015a, 2016).

*Marinomonas* and *Pseudoalteromonas*, taxa typically found in marine biofilms (Lawes et al., 2016), were only observed in SSW biofilms, the former in oil and dispersant treatments, and the latter in all treatments. *Marinomonas* are potential oil degraders (Brakstad et al., 2008) and *Pseudoalteromonas* have been implicated in hydrocarbon degradation in the water column (Gutierrez et al., 2013); however, the dominance of this taxon in both SSW control and treatment groups suggests that it may also have other roles in biofilm communities on carbon steel. *Halomonas*, which was abundant in all control and treatment groups in both experiments, has also been implicated in both EPS production and hydrocarbon degradation (Arnosti et al., 2016). The presence of these OTUs in metagenome taxonomy in this study corroborates results of biofilm 16S taxonomy data sourced from SSW in a previous study (Salerno et al., 2018), which demonstrated the prevalence of taxa associated with biofilm formation. To fully understand the impacts of contaminant exposure on developing biofilms, functional responses must also be evaluated in tandem with compositional shifts (Galand et al., 2018), which was accomplished in the current study through analysis of the metagenome.

Although community compositions between SSW and DSW biofilms had limited similarity (Supplementary Table 4), the functional profiles of all metagenomes were at least 60–70% similar to each other (Figure 5 and Supplementary Table 5). It can be difficult to quantitate functional redundancy within microbial communities and currently there are no widely accepted methods. In this work, we used CLUSTER and NMDS plots to visualize major functional differences of highly abundant proteins mapped to functional hierarchies to help decipher functional redundancy within these communities. SIMPER analysis of these abundant KOs that determined similarity between SSW and DSW metagenomes was driven primarily by translation KOs and protein families. Signaling and cellular processes KOs were also the most abundant functional categories across all metagenomes (Figure 4). Although information about specific functions in marine biofilms is still emerging, protein translation is known to be an essential function during the transition from planktonic cells to surface-attached cells during biofilm formation (Qayyum et al., 2016). Cellular signaling within biofilms, particularly quorum sensing, is also known to play an important role in the biofilm formation process (Dang and Lovell, 2016; Wang et al., 2020). The prevalence of these functional groups in all metagenomes, including treatments, indicates that biofilms observed in this study were undergoing development, despite being exposed to contaminants, and can be considered functionally redundant in this regard.

The analysis of the most abundant KOs also showed that the SSW oil and dispersant metagenomes grouped with all DSW metagenomes while SSW control and dispersed oil metagenomes clustered separately (Figure 5). SIMPER (on abundant KOs) revealed that differences between SSW and DSW metagenomes were driven by membrane transport KOs, found in higher abundance in all DSW treatment groups relative to SSW. Membrane transport genes are important to particle-associated microbes in marine environments (Dang and Lovell, 2016), specifically for their role in transporting siderophores through the cellular membrane, which bind iron for energy (Schalk et al., 2012). This may be vital for marine biofilms growing on carbon steel, although it is unclear why DSW metagenomes have higher abundance of membrane transport KOs than SSW metagenomes. DSW metagenomes also had a higher abundance of cell motility proteins, including three chemotaxis proteins, which were highest in the DSW control and lowest in the SSW dispersed oil, suggesting that exposure to dispersed oil in SSW may impede bacterial movement, which is a crucial function in developing biofilms (Oliveira et al., 2016).

The MaAsLin analysis of all KOs provides the most direct comparison of metagenome functional data across the DSW and SSW experiments because it enables the treatment groups to be analyzed individually. The MaAsLin2 control comparison revealed that over 90% of gene associations were significantly different (Table 1). This value declined below 50% in all other treatment comparisons, with the lowest dissimilarity (46%) between the SSW and DSW dispersed oil treatments. These data suggests that exposure to oil and dispersant in this experiment resulted in greater functional redundancy than in communities not challenged by these compounds. This is a significant outcome of this work, as it shows that while functional redundancy was generally evident across two experiments having different source communities, greater redundancy may emerge as a consequence of challenging environmental conditions.

Regardless of receiving the same treatment effects, under the same experimental conditions, taxonomically distinct metagenomes emerged between SSW and DSW experiments, likely as a result of different source water environmental conditions that dictated the phylogeny of communities present. This confirms prior amplicon sequencing-based studies of marine microbiome responses to the *Deepwater Horizon* spill (Hamdan et al., 2018; Salerno et al., 2018; Mugge et al., 2019a). Advances in sequencing technology have permitted insight into the metabolic profiles of microorganisms and how taxonomically distinct groups can share common functions within an ecosystem (Rosenfeld, 2002). However, Galand et al. (2018) challenged functional redundancy within marine communities by demonstrating that altering microbial communities across space and time also alters their functional attributes, which further highlights the existing knowledge gap surrounding diversity and genetics of marine microorganisms. The current study and previous works (Lee et al., 2014; Lawes et al., 2016; Toyofuku et al., 2016) indicate that functional redundancy should be interpreted with caution (Fetzer et al., 2015), as multiple parameters can shape the biofilm microbiome, including interspecific interactions, inherent biodiversity, and ambient environmental conditions, and should be included in evaluations of microbial functional redundancy.

The goal of this study was to visit the topic of functional redundancy in biofilms recruited to carbon steel surfaces from different seawater source communities. Our prior 16S rRNA studies of these communities provided evidence of the taxonomically distinct nature of these communities, which was confirmed with the metagenome results presented here. However, the functional capabilities of the communities under oil spill conditions simulated in laboratory microcosms were unknown. This study demonstrates that partial functional redundancy is evident when these taxonomically distinct biofilm communities were held under the same experimental conditions. The degree of redundancy, however, was not uniform, providing evidence that exposure to crude oil and chemical dispersant may differentially impact biofilm functional redundancy. Specifically, there is less functional similarity in the absence of environmental challenges than under treatment effects. The study reinforces previous findings that the history of microbial communities will dictate their composition and functional potential (Fetzer et al., 2015; Galand et al., 2018; Louca et al., 2018). However, when marine microbiomes encounter anthropogenic contamination events, more uniform functional responses may emerge. This information may inform understanding of microbiome responses to oil spills in surface waters and in the deep sea especially when they occur proximate to steel structures, including historic shipwrecks and oil and gas infrastructure. The work may also provide better understanding of impacts oil spills may have on the microbiomes of artificial and natural reef and fouling communities. Although much remains to be discovered about functional responses in marine biofilm microbiomes, understanding how environmental effects, including exposure to anthropogenic contaminants, is a crucial step in shaping the broad understanding of observed biodiversity and function to address how human activities impact microbial ecosystems (Galand et al., 2018). Future studies incorporating the metatranscriptome of marine biofilms would also provide useful information to further explore the relationship, or lack thereof, between taxonomic composition and functional responses in biofilm assemblages.

## Data Availability Statement

The datasets presented in this study can be found in online repositories. The names of the repository/repositories and accession number(s) can be found in the article/Supplementary Material.

## Author Contributions

LH designed the study. RM and LH conducted the microbiome data analyses. LH and RM wrote the manuscript. All authors conducted the laboratory analyses and contributed to the manuscript.

## Conflict of Interest

The authors declare that the research was conducted in the absence of any commercial or financial relationships that could be construed as a potential conflict of interest.

## Footnotes

1 https://erma.noaa.gov/gulfofmexico/erma.html

## References

[B1] Allison S. D. Martiny J. B. (2008). Resistance, resilience, and redundancy in microbial communities. *Proc. Natl. Acad. Sci. U.S.A.* 105 11512–11519. 10.1073/pnas.0801925105 18695234 PMC2556421

[B2] Andrews S. (2010). *FastQC: A Quality Control Tool for High Throughput Sequence Data.* Burlington, MA: ScienceOpen, Inc.

[B3] Arnosti C. Ziervogel K. Yang T. Teske A. (2016). Oil-derived marine aggregates–hot spots of polysaccharide degradation by specialized bacterial communities. *Deep Sea Res. Part II Top. Stud. Oceanogr.* 129 179–186. 10.1016/j.dsr2.2014.12.008

[B4] Bienhold C. Zinger L. Boetius A. Ramette A. (2016). Diversity and biogeography of bathyal and abyssal seafloor bacteria. *PLoS One* 11:e0148016. 10.1371/journal.pone.0148016 26814838 PMC4731391

[B5] Bolger A. M. Lohse M. Usadel B. (2014). Trimmomatic: a flexible trimmer for Illumina sequence data. *Bioinformatics* 30 2114–2120. 10.1093/bioinformatics/btu170 24695404 PMC4103590

[B6] Brakstad O. G. Nonstad I. Faksness L.-G. Brandvik P. J. (2008). Responses of microbial communities in Arctic sea ice after contamination by crude petroleum oil. *Microb. Ecol.* 55 540–552. 10.1007/s00248-007-9299-x 17805918

[B7] Burke C. Steinberg P. Rusch D. Kjelleberg S. Thomas T. (2011). Bacterial community assembly based on functional genes rather than species. *Proc. Natl. Acad. Sci. U.S.A.* 108 14288–14293. 10.1073/pnas.1101591108 21825123 PMC3161577

[B8] Chronopoulou P. M. Sanni G. O. Silas-Olu D. I. Van Der Meer J. R. Timmis K. N. Brussaard C. P. (2015). Generalist hydrocarbon-degrading bacterial communities in the oil-polluted water column of the North Sea. *Microb. Biotechnol.* 8 434–447. 10.1111/1751-7915.12176 25251384 PMC4408176

[B9] Comeau A. M. Douglas G. M. Langille M. G. I. (2017). Microbiome helper: a custom and streamlined workflow for microbiome research. *mSystems* 2 e127–e116. 10.1128/mSystems.00127-16 PMC5209531 28066818

[B10] Dang H. Lovell C. R. (2016). Microbial surface colonization and biofilm development in marine environments. *Microbiol. Mol. Biol. Rev.* 80 91–138. 10.1128/mmbr.00037-15 26700108 PMC4711185

[B11] Dobretsov S. Rittschof D. (2020). Love at first taste: induction of larval settlement by marine microbes. *Int. J. Mol. Sci.* 21:731. 10.3390/ijms21030731 31979128 PMC7036896

[B12] Elifantz H. Horn G. Ayon M. Cohen Y. Minz D. (2013). Rhodobacteraceae are the key members of the microbial community of the initial biofilm formed in Eastern Mediterranean coastal seawater. *FEMS Microbiol. Ecol.* 85 348–357. 10.1111/1574-6941.12122 23551015

[B13] Fetzer I. Johst K. Schäwe R. Banitz T. Harms H. Chatzinotas A. (2015). The extent of functional redundancy changes as species’ roles shift in different environments. *Proc. Natl. Acad. Sci. U.S.A.* 112 14888–14893. 10.1073/pnas.1505587112 26578806 PMC4672811

[B14] Forth H. P. Mitchelmore C. L. Morris J. M. Lipton J. (2017). Characterization of oil and water accommodated fractions used to conduct aquatic toxicity testing in support of the Deepwater Horizon oil spill natural resource damage assessment. *Environ. Toxicol. Chem.* 36 1450–1459. 10.1002/etc.3672 27805278

[B15] Franzosa E. A. Mciver L. J. Rahnavard G. Thompson L. R. Schirmer M. Weingart G. (2018). Species-level functional profiling of metagenomes and metatranscriptomes. *Nat. Methods* 15:962. 10.1038/s41592-018-0176-y 30377376 PMC6235447

[B16] Gage J. D. (2004). Diversity in deep-sea benthic macrofauna: the importance of local ecology, the larger scale, history and the Antarctic. *Deep Sea Res. Part II Top. Stud. Oceanogr.* 51 1689–1708. 10.1016/j.dsr2.2004.07.013

[B17] Galand P. E. Pereira O. Hochart C. Auguet J. C. Debroas D. (2018). A strong link between marine microbial community composition and function challenges the idea of functional redundancy. *ISME J.* 12 2470–2478. 10.1038/s41396-018-0158-1 29925880 PMC6155072

[B18] Gao X. Gao W. Cui Z. Han B. Yang P. Sun C. (2015). Biodiversity and degradation potential of oil-degrading bacteria isolated from deep-sea sediments of South Mid-Atlantic Ridge. *Mar. Pollut. Bull.* 97 373–380. 26077158 10.1016/j.marpolbul.2015.05.065

[B19] Gontikaki E. Potts L. Anderson J. Witte U. (2018). Hydrocarbon-degrading bacteria in deep-water subarctic sediments (Faroe-Shetland Channel). *J. Appl. Microbiol.* 125 1040–1053. 10.1111/jam.14030 29928773 PMC6849767

[B20] Grimaud R. Ghiglione J.-F. Cagnon C. Lauga B. Vaysse P.-J. Rodriguez-Blanco A. (2012). Genome sequence of the marine bacterium *Marinobacter hydrocarbonoclasticus* SP17, which forms biofilms on hydrophobic organic compounds. *Am. Soc. Microbiol.* 194 3539–3540. 10.1128/jb.00500-12 22689231 PMC3434751

[B21] Grzegorczyk M. Pogorzelski S. J. Pospiech A. Boniewicz-Szmyt K. (2018). Monitoring of marine biofilm formation dynamics at submerged solid surfaces with multitechnique sensors. *Front. Mar. Sci.* 5:363. 10.3389/fmars.2018.00363

[B22] Gutierrez T. Singleton D. R. Berry D. Yang T. Aitken M. D. Teske A. (2013). Hydrocarbon-degrading bacteria enriched by the Deepwater Horizon oil spill identified by cultivation and DNA-SIP. *ISME J.* 7 2091–2104. 10.1038/ismej.2013.98 23788333 PMC3806270

[B23] Hadfield M. G. (2011). Biofilms and marine invertebrate larvae: what bacteria produce that larvae use to choose settlement sites. *Annu. Rev. Mar. Sci.* 3 453–470. 10.1146/annurev-marine-120709-142753 21329213

[B24] Hamdan L. J. Coffin R. B. Sikaroodi M. Greinert J. Treude T. Gillevet P. M. (2013). Ocean currents shape the microbiome of Arctic marine sediments. *ISME J.* 7 685–696. 10.1038/ismej.2012.143 23190727 PMC3603395

[B25] Hamdan L. J. Salerno J. L. Reed A. Joye S. B. Damour M. (2018). The impact of the Deepwater Horizon blowout on historic shipwreck-associated sediment microbiomes in the northern Gulf of Mexico. *Sci. Rep.* 8:9057. 29955123 10.1038/s41598-018-27350-z PMC6023898

[B26] Handley K. M. Lloyd J. R. (2013). Biogeochemical implications of the ubiquitous colonization of marine habitats and redox gradients by *Marinobacter* species. *Front. Microbiol.* 4:136. 10.3389/fmicb.2013.00136 23734151 PMC3660661

[B27] Hu P. Dubinsky E. A. Probst A. J. Wang J. Sieber C. M. Tom L. M. (2017). Simulation of Deepwater Horizon oil plume reveals substrate specialization within a complex community of hydrocarbon degraders. *Proc. Natl. Acad. Sci. U.S.A.* 114 7432–7437. 10.1073/pnas.1703424114 28652349 PMC5514739

[B28] Hwang C. Y. Zhang G. I. Kang S.-H. Kim H. J. Cho B. C. (2009). *Pseudomonas* pelagia sp. nov., isolated from a culture of the Antarctic green alga *Pyramimonas gelidicola*. *Int. J. Syst. Evol. Microbiol.* 59 3019–3024. 10.1099/ijs.0.008102-0 19643900

[B29] Kanehisa M. Goto S. (2000). KEGG: kyoto encyclopedia of genes and genomes. *Nucleic Acids Res.* 28 27–30. 10592173 10.1093/nar/28.1.27 PMC102409

[B30] Kaplan J. Á (2010). Biofilm dispersal: mechanisms, clinical implications, and potential therapeutic uses. *J. Dent. Res.* 89 205–218. 10.1177/0022034509359403 20139339 PMC3318030

[B31] Kleindienst S. Grim S. Sogin M. Bracco A. Crespo-Medina M. Joye S. B. (2016). Diverse, rare microbial taxa responded to the Deepwater Horizon deep-sea hydrocarbon plume. *ISME J.* 10 400–415. 10.1038/ismej.2015.121 26230048 PMC4737931

[B32] Kleindienst S. Paul J. H. Joye S. B. (2015a). Using dispersants after oil spills: impacts on the composition and activity of microbial communities. *Nat. Rev. Microbiol.* 13 388–396. 10.1038/nrmicro3452 25944491

[B33] Kleindienst S. Seidel M. Ziervogel K. Grim S. Loftis K. Harrison S. (2015b). Chemical dispersants can suppress the activity of natural oil-degrading microorganisms. *Proc. Natl. Acad. Sci. U.S.A.* 112 14900–14905. 10.1073/pnas.1507380112 26553985 PMC4672791

[B34] Kviatkovski I. Minz D. (2015). A member of the Rhodobacteraceae promotes initial biofilm formation via the secretion of extracellular factor (s). *Aquat. Microb. Ecol.* 75 155–167. 10.3354/ame01754

[B35] Langmead B. Salzberg S. L. (2012). Fast gapped-read alignment with Bowtie 2. *Nat. Methods* 9 357–359. 10.1038/nmeth.1923 22388286 PMC3322381

[B36] Lawes J. C. Neilan B. A. Brown M. V. Clark G. F. Johnston E. L. (2016). Elevated nutrients change bacterial community composition and connectivity: high throughput sequencing of young marine biofilms. *Biofouling* 32 57–69. 10.1080/08927014.2015.1126581 26751559

[B37] Lee J. Ray R. Lemieux E. Falster A. Little B. (2004). An evaluation of carbon steel corrosion under stagnant seawater conditions. *Biofouling* 20 237–247. 10.1080/08927010400013274 15621645

[B38] Lee J. S. Ray R. I. Little B. J. Lemieux E. (2005). Evaluation of deoxygenation as a corrosion control measure for ballast tanks. *Corrosion* 61 1173–1188. 10.5006/1.3278153

[B39] Lee O. O. Wang Y. Tian R. Zhang W. Shek C. S. Bougouffa S. (2014). In situ environment rather than substrate type dictates microbial community structure of biofilms in a cold seep system. *Sci. Rep.* 4:3587. 24399144 10.1038/srep03587 PMC5378041

[B40] Lema K. A. Constancias F. Rice S. A. Hadfield M. G. (2019). High bacterial diversity in nearshore and oceanic biofilms and their influence on larval settlement by Hydroides elegans (Polychaeta). *Environ. Microbiol.* 21 3472–3488. 10.1111/1462-2920.14697 31136079

[B41] Louca S. Polz M. F. Mazel F. Albright M. B. Huber J. A. O’connor M. I. (2018). Function and functional redundancy in microbial systems. *Nat. Ecol. Evol.* 1 936–943. 10.1038/s41559-018-0519-1 29662222

[B42] Mallick H. Ma S. Franzosa E. A. Vatanen T. Morgan X. C. Huttenhower C. (2017). Experimental design and quantitative analysis of microbial community multiomics. *Genome Biol.* 18:228. 29187204 10.1186/s13059-017-1359-z PMC5708111

[B43] McBeth J. M. Little B. J. Ray R. I. Farrar K. M. Emerson D. (2011). Neutrophilic iron-oxidizing “Zetaproteobacteria” and mild steel corrosion in nearshore marine environments. *Appl. Environ. Microbiol.* 77 1405–1412. 10.1128/aem.02095-10 21131509 PMC3067224

[B44] McKay L. J. Gutierrez T. Teske A. P. (2016). Development of a group-specific 16S rRNA-targeted probe set for the identification of *Marinobacter* by fluorescence in situ hybridization. *Deep Sea Res. Part II Top. Stud. Oceanogr.* 129 360–367. 10.1016/j.dsr2.2013.10.009

[B45] Mounier J. Camus A. Mitteau I. Vaysse P.-J. Goulas P. Grimaud R. (2014). The marine bacterium *Marinobacter hydrocarbonoclasticus* SP17 degrades a wide range of lipids and hydrocarbons through the formation of oleolytic biofilms with distinct gene expression profiles. *FEMS Microbiol. Ecol.* 90 816–831. 10.1111/1574-6941.12439 25318592

[B46] Mugge R. L. Brock M. L. Salerno J. L. Damour M. Church R. A. Lee J. (2019a). Deep sea biofilms, historic shipwreck preservation and the Deepwater Horizon spill. *Front. Mar. Sci.* 6:48. 10.3389/fmars.2019.00048

[B47] Mugge R. L. Lee J. S. Brown T. T. Hamdan L. J. (2019b). Marine biofilm bacterial community response and carbon steel loss following Deepwater Horizon spill contaminant exposure. *Biofouling* 35 870–882. 10.1080/08927014.2019.1673377 31603038

[B48] Oliveira N. M. Foster K. R. Durham W. M. (2016). Single-cell twitching chemotaxis in developing biofilms. *Proc. Natl. Acad. Sci. U.S.A.* 113 6532–6537. 10.1073/pnas.1600760113 27222583 PMC4988597

[B49] Orland C. Emilson E. J. Basiliko N. Mykytczuk N. C. Gunn J. M. Tanentzap A. J. (2019). Microbiome functioning depends on individual and interactive effects of the environment and community structure. *ISME J.* 13 1–11. 10.1038/s41396-018-0230-x 30042502 PMC6298968

[B50] Qayyum S. Sharma D. Bisht D. Khan A. U. (2016). Protein translation machinery holds a key for transition of planktonic cells to biofilm state in *Enterococcus faecalis*: a proteomic approach. *Biochem. Biophys. Res. Commun.* 474 652–659. 10.1016/j.bbrc.2016.04.145 27144316

[B51] Qi W. Colarusso A. Olombrada M. Parrilli E. Patrignani A. Tutino M. L. (2019). New insights on *Pseudoalteromonas haloplanktis* TAC125 genome organization and benchmarks of genome assembly applications using next and third generation sequencing technologies. *Sci. Rep.* 9:16444. 31712730 10.1038/s41598-019-52832-z PMC6848147

[B52] Rosenfeld J. S. (2002). Functional redundancy in ecology and conservation. *Oikos* 98 156–162. 10.1034/j.1600-0706.2002.980116.x 11841302

[B53] Salerno J. L. Little B. Lee J. Hamdan L. J. (2018). Exposure to crude oil and chemical dispersant may impact marine microbial biofilm composition and steel corrosion. *Front. Mar. Sci.* 5:196. 10.3389/fmars.2018.00196

[B54] Salta M. Wharton J. A. Blache Y. Stokes K. R. Briand J. F. (2013). Marine biofilms on artificial surfaces: structure and dynamics. *Environ. Microbiol.* 15 2879–2893. 23869714 10.1111/1462-2920.12186

[B55] Schalk I. J. Mislin G. L. Brillet K. (2012). Structure, function and binding selectivity and stereoselectivity of siderophore–iron outer membrane transporters. *Curr. Top. Membr.* 69 37–66. 10.1016/b978-0-12-394390-3.00002-1 23046646

[B56] Techtmann S. M. Fitzgerald K. S. Stelling S. C. Joyner D. C. Uttukar S. M. Harris A. P. (2016). *Colwellia psychrerythraea* strains from distant deep sea basins show adaptation to local conditions. *Front. Environ. Sci.* 4:33. 10.3389/fenvs.2016.00033

[B57] Toyofuku M. Inaba T. Kiyokawa T. Obana N. Yawata Y. Nomura N. (2016). Environmental factors that shape biofilm formation. *Biosci. Biotechnol. Biochem.* 80 7–12. 10.1080/09168451.2015.1058701 26103134

[B58] Tremblay J. Yergeau E. Fortin N. Cobanli S. Elias M. King T. L. (2017). Chemical dispersants enhance the activity of oil-and gas condensate-degrading marine bacteria. *ISME J.* 11:2793. 10.1038/ismej.2017.129 28800137 PMC5702735

[B59] Truong D. T. Franzosa E. A. Tickle T. L. Scholz M. Weingart G. Pasolli E. (2015). MetaPhlAn2 for enhanced metagenomic taxonomic profiling. *Nat. Methods* 12:902. 10.1038/nmeth.3589 26418763

[B60] Valentine D. L. Fisher G. B. Bagby S. C. Nelson R. K. Reddy C. M. Sylva S. P. (2014). Fallout plume of submerged oil from Deepwater Horizon. *Proc. Natl. Acad. Sci. U.S.A.* 111 15906–15911. 10.1073/pnas.1414873111 25349409 PMC4234598

[B61] Wang R. Ding W. Long L. Lan Y. Tong H. Saha S. (2020). Exploring the influence of signal molecules on marine biofilms development. *Front. Microbiol.* 11:571400. 10.3389/fmicb.2020.571400 33281767 PMC7691533

[B62] Zhang J. Kobert K. Flouri T. Stamatakis A. (2013). PEAR: a fast and accurate Illumina Paired-End reAd mergeR. *Bioinformatics* 30 614–620. 10.1093/bioinformatics/btt593 24142950 PMC3933873

[B63] Zhang W. Ding W. Li Y.-X. Tam C. Bougouffa S. Wang R. (2019). Marine biofilms constitute a bank of hidden microbial diversity and functional potential. *Nat. Commun.* 10:517. 30705275 10.1038/s41467-019-08463-z PMC6355793

