# Supplementary material for: Microbial Functional Responses in Marine Biofilms Exposed to Deepwater Horizon Spill Contaminants
Source: Front Microbiol. 2021 Feb 25;12:636054. doi: 10.3389/fmicb.2021.636054 (PMC7947620; doi:10.3389/fmicb.2021.636054)
Supplement: Supplementary file 8 [file Image_1.PDF]

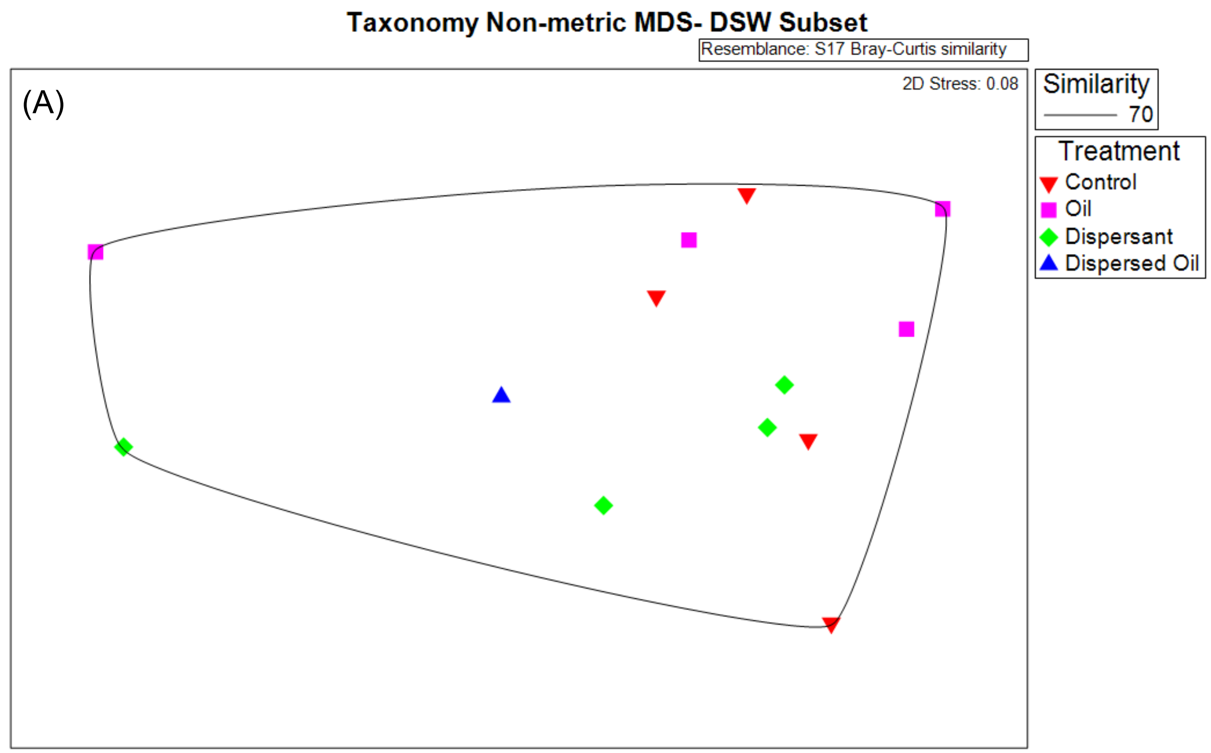

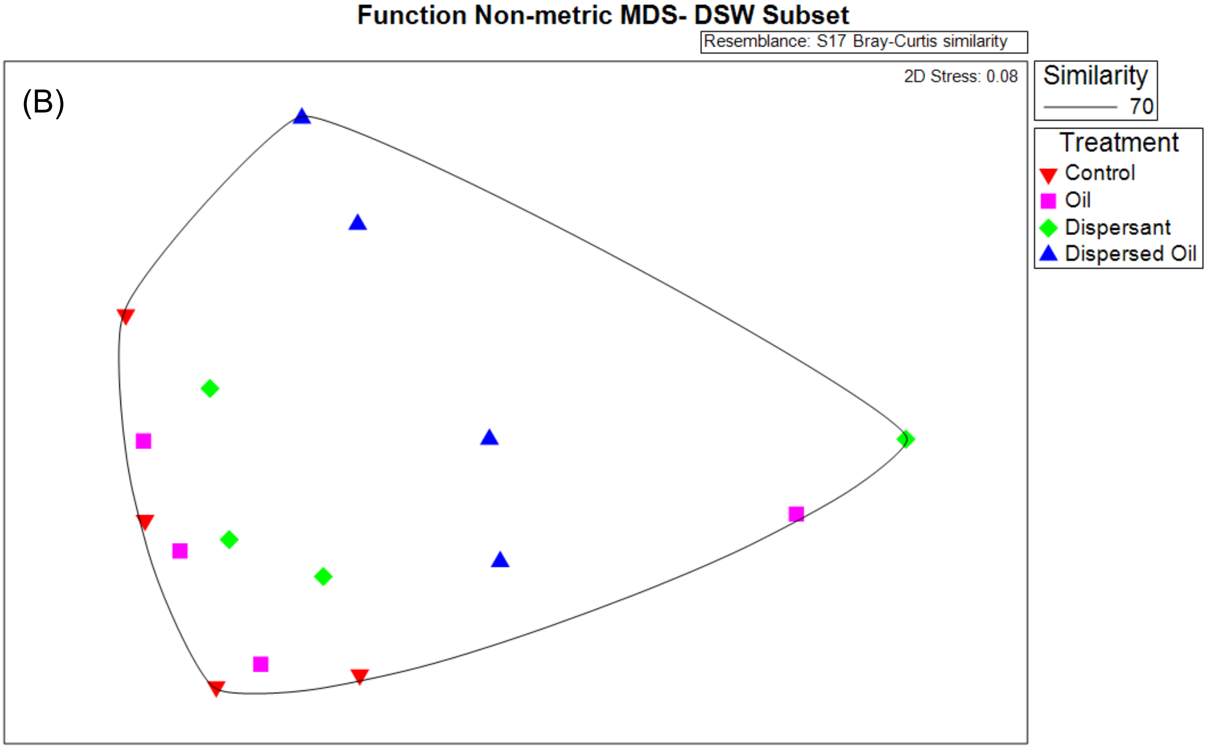

Supplementary Figure S1. Non-metric multidimensional scaling (NMDS) subset plots of DSW cluster from Metaphlan2 species relative abundances of taxa and (B) Humann2 results of KEGG orthologs (KOs) in copies per million (CPM) based on Bray-Curtis dissimilarity matrices calculated within PRIMER. Icons represent metagenome samples color coded by treatment: control samples in red, oil samples in pink, dispersant samples in green, and dispersed oil samples in dark blue.
