# Supplementary figures and images for: Microbial Functional Responses in Marine Biofilms Exposed to Deepwater Horizon Spill Contaminants
Source: Front Microbiol. 2021 Feb 25;12:636054. doi: 10.3389/fmicb.2021.636054 (PMC7947620; doi:10.3389/fmicb.2021.636054)

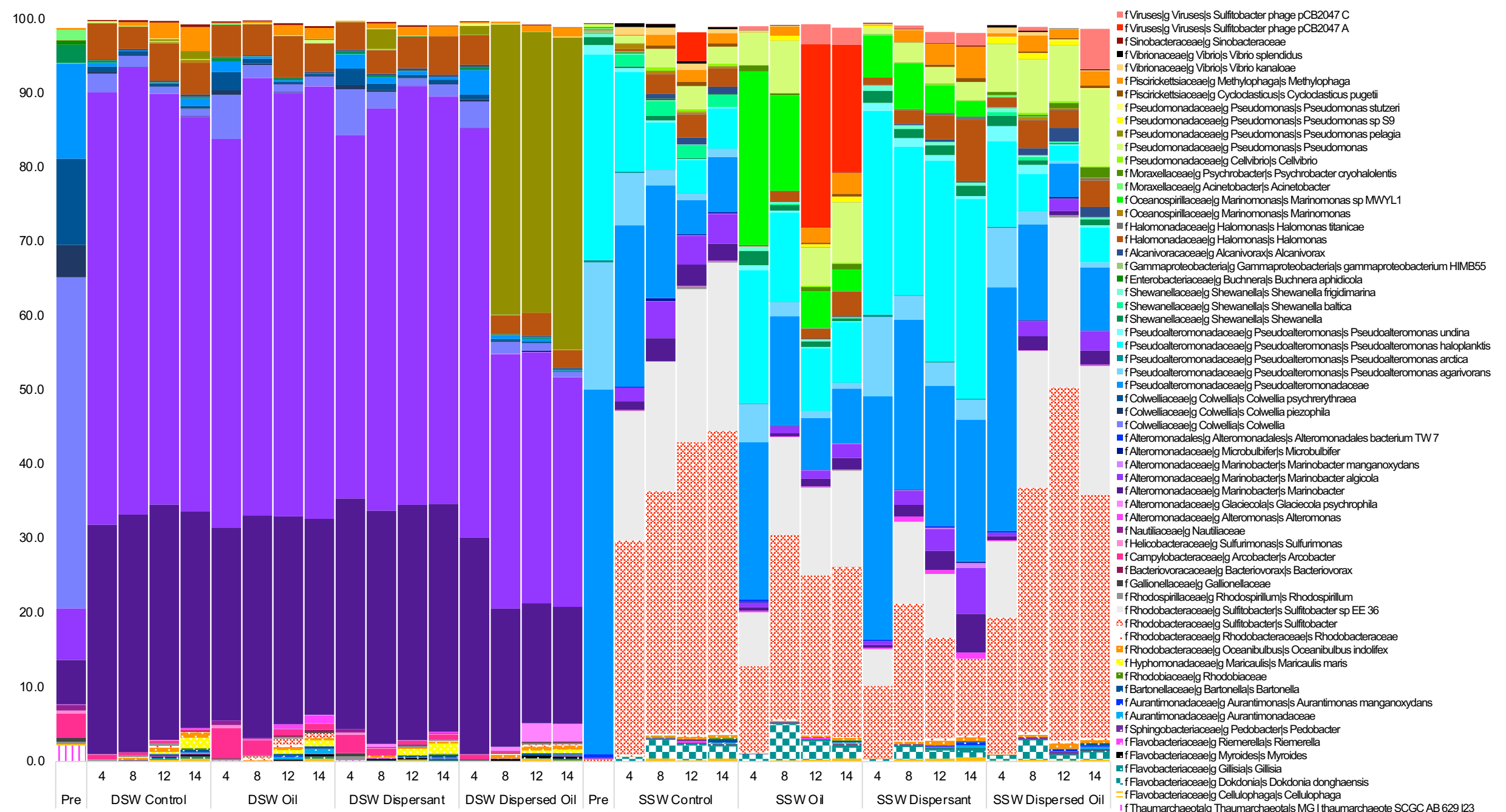

Supplement: Supplementary file 9 [file Image_2.PDF]
